# Physician‐office vs home uptake of colorectal cancer screening using FOBT/FIT among screening‐eligible US adults

**DOI:** 10.1002/cam4.2604

**Published:** 2019-10-21

**Authors:** Onyema Greg Chido‐Amajuoyi, Anushree Sharma, Rajesh Talluri, Irene Tami‐Maury, Sanjay Shete

**Affiliations:** ^1^ Department of Epidemiology The University of Texas MD Anderson Cancer Center Houston TX USA; ^2^ Department of Behavioral Sciences The University of Texas MD Anderson Cancer Center Houston TX USA; ^3^ Department of Data Science The University of Mississippi Medical Center Jackson MS USA; ^4^ Division of Cancer Prevention and Population Sciences The University of Texas MD Anderson Cancer Center Houston TX USA; ^5^ Department of Biostatistics The University of Texas MD Anderson Cancer Center Houston TX USA

**Keywords:** cancer screening, colorectal cancer, disparities, fecal occult blood test (FOBT)/fecal immunochemical test (FIT)

## Abstract

**Background:**

Guidelines of the American Cancer Society and US Preventive Services Task Force specify that colorectal cancer (CRC) screening using guaiac‐based fecal occult blood test (FOBT)/fecal immunochemical test (FIT) should be done at home. We therefore examined the prevalence and correlates of CRC screening using FOBT/FIT in physicians' office vs at home.

**Methods:**

Analysis of 9493 respondents 50‐75 years old from the Cancer Control Supplement of the 2015 National Health Interview Survey was conducted. Weighted multivariable logistic regression was used to identify the determinants of in‐office vs home use of FOBT/FIT for CRC screening.

**Results:**

Of the overall sample of screening‐eligible adults (n = 9403), only 937 (10.4%) respondents underwent CRC screening using FOBT/FIT within the past year; among this screening population, 279 (28.3%) respondents were screened in‐office. We found that sociodemographic factors alone, not CRC risk factors, determined whether FOBT/FIT would be used in‐office or at home. Hispanics had greater odds of being screened in‐office using FOBT/FIT (aOR: 2.04; 95% CI: 1.05‐3.99). Compared with those 50‐59 years old, respondents 70‐75 years old were less likely to be screened in‐office using FOBT/FIT (aOR: 0.44, 95% CI: 0.25‐0.79). Similarly, individuals residing in the Western region of the country had lower odds of in‐office FOBT/FIT (aOR: 0.26; 95% CI: 0.11‐0.58).

**Conclusion:**

Amid low overall uptake rates of FOBT/FIT in the United States, in‐physician office testing is high, indicative of a missed opportunity for effective screening and poor adherence of physicians to national guidelines. Sociodemographic factors are determinants of uptake of FOBT/FIT at home or in‐office and should be considered in designing interventions aimed at providers and the general population.

## Abstract 2

Amid low overall uptake rates of FOBT/FIT in the United States, in‐physician office testing is high, indicative of a missed opportunity for effective screening and poor adherence of physicians to national guidelines.

## Graphical abstract

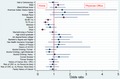

## 1 INTRODUCTION

Colorectal cancer (CRC) is the third most commonly diagnosed cancer in men and women in the United States (US).^1^, ^2^ An estimated 140 250 new cases and 50 630 deaths were attributed to CRC in 2018.^3^ When CRC is detected at an early, localized stage, the 5‐year survival rate is around 90%, whereas the survival rate is just 14% if CRC is detected after spread to a distant organ.^3^ The US Preventive Services Task Force (USPSTF) recommends CRC screening, using a variety of approaches, from ages 50 through 75 years.^4^ These approaches include yearly stool‐based tests—the guaiac‐based fecal occult blood test (FOBT) or fecal immunochemical test (FIT)—and endoscopic tests such as colonoscopy every 10 years or flexible sigmoidoscopy every 5 years.^4^

While the benefits of CRC screening are well established, it remains underutilized in the US population.^5^ Data from 2015 reveal that the overall prevalence of CRC screening in the US is 62.4%, about 8% shy of the Healthy People 2020 target of 70.5%.^5^, ^6^ While rates of colonoscopy have increased over the years, screening using FOBT steadily declined between 2008 and 2015.^7^ The poor uptake of FOBT/FIT compared with colonoscopy remains a public health conundrum, given the inherent advantages of FOBT/FIT such as its affordability, which makes it particularly attractive to individuals of low socioeconomic status,^8^, ^9^ as well as its noninvasive nature.^10^

National guidelines (including those of the USPSTF and American Cancer Society) are explicit in specifying that CRC screening using FOBT/FIT should be done at home.^4^, ^11^ Moreover several randomized trials point to the efficacy of home‐based FOBT for CRC screening.^12^, ^13^, ^14^, ^15^ A longitudinal study by Collins et al reported that in‐office FOBT had a sensitivity of only 4.9%, compared with 23.9% with home‐based FOBT.^16^ The higher sensitivity of the home‐based test has been largely attributed to the ability to collect multiple stool samples over a 3‐day period, compared with an in‐office test, which allows for collection of a single sample.^16^

Despite the proven superiority of home‐based testing, many health care providers continue to carry out CRC screening using in‐office FOBT.^17^ Nadel et al found that 32.5% of physicians in 2005 and 25% of physicians in 2010 used in‐office FOBT to screen for CRC.^18^, ^19^ In another study, 64% of obstetricians/gynecologists reported performing in‐office FOBT exclusively.^20^ Physicians may find in‐office screening attractive for a number of reasons. In addition to obtaining results almost immediately, in‐office testing boosts compliance. A study found that 81% of clinicians believed that patients who received a FIT order may not have the motivation to return home‐test kits, 61% believed that patients may forget to return home‐test kits, and 55% believed that poor understanding of test instructions was a barrier to returning home‐test kits.^21^ While physicians may conduct in‐office FOBT/FIT in a bid to address these perceived barriers and ensure their patient is screened, this practice is guideline incongruent^4^, ^11^ and deemed inadequate for CRC screening by medical bodies such as the American College of Obstetricians and Gynecologists.^22^ Other drivers of in‐office screening may include factors such as financial incentives, as well as screening in an effort to satisfy quality measures such as the Healthcare Effectiveness Data and Information Set.

Disparities exist in the incidence and mortality rates of CRC,^2^, ^23^ with rates 20% and 40% higher, respectively, in non‐Hispanic blacks than in non‐Hispanic whites. Furthermore, CRC incidence and mortality rates are 30% and 40% higher, respectively, in men than in women.^2^ These observed disparities may, in part, be attributed to differences in rates of CRC screening. Several sociodemographic factors have been found to be associated with CRC screening uptake,^24^, ^25^ with less‐educated individuals, lower‐income individuals, recent immigrants, and those without access to health care shown to have the lowest levels of CRC screening.^7^, ^26^, ^27^, ^28^ In addition, disparities have also been described in CRC screening among residents in rural versus urban areas.^29^, ^30^

While disparities have been broadly described in the use of FOBT for CRC screening, to the best of our knowledge, no previous study has investigated whether individuals' sociodemographic characteristics determine uptake of CRC screening using FOBT/FIT at home or in‐office. Given that regional^31^ and national surveys^18^, ^19^, ^20^ of physicians consistently describe a high prevalence of in‐office screening, we hypothesized that a significant proportion of participants who had used FOBT/FIT for CRC screening were tested in‐office. Second, we hypothesized that sociodemographic characteristics of individuals play a key role in determining in‐office versus home use of FOBT/FIT for CRC screening. To test our hypotheses, we analyzed a nationally representative sample of eligible US adults who had undergone CRC screening using FOBT/FIT within the past year to determine the prevalence and correlates of in‐office versus home use of FOBT/FIT for CRC screening.

## 2 MATERIALS AND METHODS

### 2.1 Study sample

Data for this study were collected from the National Health Interview Survey (NHIS), which is administered annually by the National Center for Health Statistics and samples the noninstitutionalized population of the US. Every 5 years, the NHIS administers a Cancer Control Supplement (CCS) questionnaire, which collects detailed measures on cancer screening and associated behaviors. Primary study outcome variables were obtained from the 2015 CCS. The response rate for the sample adult component of the 2015 NHIS survey was 55.2%.^32^ NHIS is approved by the Research Ethics Review Board of the National Center for Health Statistics. All NHIS respondents provided oral consent prior to participation in the survey. Additional details regarding the NHIS survey can be found at https://www.cdc.gov/nchs/nhis/index.htm.

Corresponding with USPSTF grade A recommendations for CRC screening using FOBT/FIT, our analytic sample was restricted to adults 50‐75 years old (n = 13 287). Since our study's purpose was to examine adults who used FOBT/FIT for screening purposes only, we restricted the sample to those whose main reason for testing was “routine examination” and excluded those whose main reason for carrying out FOBT/FIT at home (n = 733) or in‐office (n = 424) was “because of a problem” or “other.” Because our study focused on comparing differences in uptake of FOBT/FIT by setting (in‐office vs home), we excluded individuals who reported FOBT/FIT testing in both settings (n = 603). Furthermore, we excluded individuals reporting a personal history of CRC (n = 84). Among those who had used FOBT/FIT, in pursuance of a guideline‐compliant sample, we excluded those who had not been screened within the past year (in‐office, n = 451; home, n = 1518; see full description below). We therefore arrived at a final sample size of 9493 eligible adults.

### 2.2 Outcome measures

The primary study outcome was use of FOBT/FIT, at home or in‐office. Use of the FOBT/FIT at home or in‐office was ascertained by the following questions, respectively: (a) “Have you EVER HAD a blood stool or FIT test, using a HOME test kit?”; (b) “Have you EVER HAD a blood stool or FIT test in which your doctor or other health care professional collected a stool sample during an office visit?” Responses were categorized as “yes” or “no.” Individuals who did not respond or answered “refused,” “not ascertained,” or “don't know” were not included in the analysis. Respondents who answered “no” to both questions were considered non‐users of FOBT/FIT. Among those who reported “yes” to screening at home or in‐office, we assessed for FOBT/FIT guideline compliance using the questions: “When did you have your MOST RECENT blood stool or FIT test using a kit at HOME?” and “When did you have your MOST recent OFFICE blood stool or FIT test?” Time categories for the most recent blood stool test (using the NHIS 2000 method) were classified as: “A year ago or less,” “More than 1 year but not more than 2 years,” “More than 2 years but not more than 3 years,” “More than 3 years but not more than 5 years,” “More than 5 years but not more than 10 years,” “Over 10 years ago,” “Refused,” “Not ascertained,” and “Don't know.” The study was restricted to respondents who carried out screening “a year ago or less” (deemed USPSTF recommendation compliant).

### 2.3 Correlate measures

Correlates of FOBT/FIT for CRC screening selected for this study were informed by the literature and grouped at two levels: (a) sociodemographic characteristics^7^, ^24^ and (b) personal and familial risk factors or behaviors for CRC.^28^, ^33^, ^34^, ^35^ Sociodemographic characteristics analyzed were age, sex, marital status, level of education, geographic region, health insurance coverage status, race, and ethnicity. Region reports the region of the US where the housing unit containing survey participants was located. The four regions—Northeast, North Central/Midwest, South, and West—correspond to the US regions recognized by the Census Bureau.

CRC risk factors and behaviors analyzed were personal history of cancer other than CRC, parental history of CRC, ulcerative colitis/Crohn disease, history of polyps, alcohol drinking status, smoking status, and perceptions of CRC risk. To evaluate perception of CRC risk, respondents were asked “Compared to the average (man/woman) your age, would you say that you are more likely to get colon or rectal cancer, less likely, or about as likely?”

### 2.4 Statistical analysis

To ensure the analysis was nationally representative and reflective of the general population, we applied survey sampling weights developed for the NHIS. Weighted prevalence estimates of sociodemographic characteristics and CRC risk factors by home and in‐office FOBT/FIT CRC screening were examined. Weighted multivariable survey logistic regressions were conducted to identify associated factors. Chi‐square tests were conducted to evaluate whether subgroup differences between home and in‐office FOBT/FIT users were statistically significant. Statistical significance was defined as *P* < .05. All correlate measures used in this study had fewer than 10% missing responses.

We first conducted analyses to compare the characteristics of participants who underwent CRC screening using FOBT/FIT (regardless of setting) with those of never‐users of FOBT/FIT (Appendix). Next, we compared the characteristics of individuals who underwent CRC screening using FOBT/FIT in‐office versus home at two levels. The first level examined sociodemographic characteristics only (model 1), while the second studied both the sociodemographic factors tested in level 1 and CRC risk factors (model 2). Since previous exposure to colonoscopy or sigmoidoscopy (endoscopy) may be related to both study outcome and exposure parameters, and thus play a role in the uptake of FOBT/FIT, we controlled for endoscopy as a possible confounder by including it in all study models. All analyses were carried out using STATA, version 14.2 (Stata Corp. 2015, Stata Statistical Software, release 14).

## 3 RESULTS

### 3.1 Descriptive characteristics

Table 1 describes the distribution and weighted percentages of sociodemographic and CRC risk factors. Of the overall sample of screening‐eligible adults (n = 9403), only 937 (10.4%) respondents underwent CRC screening using FOBT/FIT within the past year; among this screening population, 279 (28.3%) respondents were screened in‐office.

**Table 1:** Distribution and weighted prevalence of FOBT/FIT uptake among US Adults (50‐75 y), by sociodemographic characteristics and CRC risk factors (N = 9493)—National Health Interview Survey, 2015

	Study sample (n)	Used FOBT/FIT a year ago or less		
		No [n = 8556 (89.6%)]	Yes [n = 937 (10.4%)]	
			In‐office [n = 279 (28.3%)]	Home [n = 658 (71.7%)]
Sex	9493			
Female	5152 (52.2%)	4657 (52.6%)	144 (47.1%)	351 (48.9%)
Male	4341 (47.8%)	3899 (47.4%)	135 (52.9%)	307 (51.1%)
Race	9480			
White	7408 (81.0%)	6693 (81.1%)	218 (78.2%)	497 (80.7%)
Black/African American	1344 (11.3%)	1216 (11.4%)	38 (11.9%)	90 (10.6%)
American Indian/Alaska Native	98 (0.96%)	81 (0.89%)	6 (2.58%)	11 (1.17%)
Asian	480 (5.54%)	419 (5.43%)	12 (5.68%)	49 (6.79%)
Multiple	150 (1.16%)	135 (1.18%)	5 (1.64%)	10 (0.72%)
Hispanic	9493			
No	8269 (88.7%)	7475 (88.9%)	233 (82.3%)	561 (88.8%)
Yes	1224 (11.3%)	1081 (11.1%)	46 (17.7%)	97 (11.2%)
Age, y	9493			
50‐59	4389 (51.1%)	4069 (52.4%)	112 (47.2%)	208 (36.8%)
60‐69	3635 (35.7%)	3232 (35.2%)	120 (39.4%)	283 (40.5%)
70‐75	1469 (13.2%)	1255 (12.4%)	47 (13.4%)	167 (22.7%)
Region	9493			
Northeast	1666 (18.8%)	1517 (19.3%)	56 (19.2%)	93 (13.5%)
Midwest	1942 (22.2%)	1813 (23.1%)	51 (18.9%)	78 (12.0%)
South	3303 (37.5%)	2999 (37.7%)	107 (44.4%)	197 (32.3%)
West	2582 (21.5%)	2227 (19.9%)	65 (17.6%)	290 (42.1%)
Marital status	9467			
Not married	4459 (32.1%)	4061 (32.7%)	123 (28.8%)	275 (26.4%)
Married/living with partner	5008 (67.9%)	4472 (67.3%)	155 (71.2%)	381 (73.6%)
Highest education	9454			
No high school diploma/ GED recipient	1673 (15.6%)	1511(15.7%)	48 (14.5%)	114 (14.5%)
High school graduate	2269 (23.4%)	2072 (23.1%)	61 (23.5%)	136 (21.0%)
AA degree/some college	2839 (29.5%)	2568 (29.6%)	71 (23.7%)	200 (29.8%)
Bachelor's degree and higher	2673 (31.5%)	2368 (31.0%)	97 (38.3%)	208 (34.7%)
Health insurance	9464			
Not covered	694 (6.82%)	666 (7.27%)	8 (1.72%)	20 (3.35%)
Covered	8779 (93.2%)	7861 (92.7%)	271 (98.3%)	638 (96.6%)
History of polyp	9459			
No	7745 (81.4)	7028 (82.1%)	199 (72.8%)	518 (75.9%)
Yes	1714 (18.6%)	1497 (17.9%)	80 (27.2%)	137 (24.1%)
Alcohol drinking status	9422			
Life time abstainer	1883 (18.1%)	1721 (18.3%)	55 (21.1%)	107 (13.7%)
Former	1973 (19.5%)	1747 (19.4%)	57 (18.4%)	169 (21.9%)
Current light‐moderate	5055 (57.0%)	4565 (57.0%)	150 (52.5%)	340 (58.0%)
Current heavy	511 (5.44%)	455 (5.27%)	17 (8.02%)	39 (6.37%)
Smoking status	9480			
Never smoker	5037 (55.1%)	4548 (55.0%)	154 (60.9%)	335 (54.2%)
Former smoker	2736 (28.7%)	2414 (28.2%)	79 (28.3%)	243 (35.5%)
Current smoker	1707 (16.2%)	1583 (16.9%)	46 (10.8%)	78 (10.3%)
Perception of CRC risk vs average person	8781			
Less likely	4035 (45.5%)	3632 (45.0%)	113 (47.1%)	290 (50.9%)
About as likely	4175 (48.3%)	3782 (48.7%)	129 (46.6%)	264 (44.0%)
More likely	571 (6.17%)	516 (6.25%)	17 (6.32%)	38 (5.04%)
Parental history of CRC	9493			
No	8940 (94.2%)	8048 (94.1%)	263 (94.5%)	629 (96.0%)
Yes	553 (5.75%)	508 (5.91%)	16 (5.47%)	29 (4.03%)
Personal history of cancer (excluding CRC)	9484			
No	8319 (87.8%)	7528 (88.3%)	234 (85.5%)	557 (82.6%)
Yes	1165 (12.2%)	1019 (11.7%)	45 (14.5%)	101 (17.4%)
History of ulcerative colitis/Crohn's disease	9477			
No	9353 (98.6%)	8431 (98.6%)	274 (96.8%)	648 (98.7%)
Yes	124 (1.42%)	109 (1.37%)	5 (3.18%)	10 (1.28%)
Previous colonoscopy/sigmoidoscopy	9486			
No	3962 (41.0%)	3665 (42.1%)	70 (25.9%)	227 (33.9%)
Yes	5524 (59.0%)	4884 (57.9%)	209 (74.1%)	431 (66.1%)

Abbreviations: CRC = colorectal cancer; FIT, fecal immunochemical test; FOBT, fecal occult blood test.

#### 3.1.1 In‐office screening

Among those who had in‐office FOBT/FIT, most were male (52.9%) and white (78.2%). About 17.7% were Hispanic. The largest proportion of respondents who reported in‐office testing was between 50 and 59 years old (47.2%) and resided in the Southern region of the country (44.4%). Most respondents (74.1%) reported having had a previous colonoscopy or sigmoidoscopy.

#### 3.1.2 At‐home screening

Among those participants who used FOBT/FIT at home, most were male (51.1%) and white (80.7%). Only 11.2% of the participants who used FOBT/FIT at home were Hispanic. The largest proportion of those who used the tests at home was between the ages of 60 and 69 (40.5%) and resided in the Western region of the country (42.1%). Most (66.1%) of those who used FOBT/FIT at home had undergone a previous colonoscopy or sigmoidoscopy.

### 3.2 In‐office versus home FOBT/FIT comparison

#### 3.2.1 Model 1 (sociodemographic)

In a multivariable regression analysis comparing in‐office versus home uptake of FOBT/FIT (Table 2), ethnicity, age, and region of residence were found to be significantly associated correlates.

**Table 2:** Multivariable logistic regression assessing the association between use of FOBT/FIT in‐office vs at home (base outcome) and sociodemographic characteristics and CRC risk factors of US adults (50‐75 y)—National Health Interview Survey, 2015

	Model 1^a^, ^b^				Model 2^a^, ^c^			
	aOR	[95% CI]		*P*	aOR	[95% CI]		*P*
Sex								
Female	Ref.				Ref.			
Male	1.10	0.73	1.66	.637	1.13	0.73	1.75	.583
Race								
White	Ref.				Ref.			
Black/African American	1.04	0.56	1.94	.902	1.03	0.54	1.97	.929
American Indian/Alaska Native	2.93	0.73	11.71	.128	3.66	0.92	14.60	.065
Asian	1.39	0.59	3.26	.452	1.41	0.56	3.54	.465
Multiple	3.27	0.79	13.58	.102	3.31	0.73	14.91	.119
Hispanic								
No	Ref.				Ref.			
Yes	2.41	1.22	4.78	**.012**	2.04	1.04	3.99	**.037**
Age, y								
50‐59	Ref.				Ref.			
60‐69	0.67	0.43	1.05	.081	0.69	0.43	1.10	.119
70‐75	0.39	0.22	0.70	**.001**	0.44	0.25	0.79	**.006**
Region								
Northeast	Ref.				Ref.			
Midwest	1.25	0.59	2.68	.560	1.44	0.65	3.17	.370
South	1.03	0.54	1.97	.929	0.98	0.49	1.95	.960
West	0.28	0.12	0.61	**.002**	0.26	0.11	0.58	**.001**
Marital status								
Not married	Ref.				Ref.			
Married/living with partner	0.80	0.53	1.20	.280	0.76	0.49	1.17	.211
Highest education								
No high school diploma/ GED recipient	Ref.				Ref.			
High school graduate	1.24	0.62	2.48	.538	1.09	0.54	2.19	.805
AA degree/some college	0.92	0.51	1.68	.796	0.93	0.48	1.80	.830
Bachelor's degree and higher	1.29	0.69	2.41	.427	1.24	0.62	2.45	.541
Health insurance								
Not covered	Ref.				Ref.			
Covered	1.09	0.55	2.18	.806	1.98	0.63	6.22	.240
History of polyps								
No					Ref.			
Yes					1.01	0.61	1.66	.979
History of ulcerative colitis/Crohn's disease								
No					Ref.			
Yes					2.72	0.79	9.41	.113
Alcohol drinking status								
Lifetime abstainer					Ref.			
Former					0.60	0.28	1.26	.175
Current (light‐moderate)					0.60	0.33	1.08	.089
Current (heavy)					0.99	0.36	2.74	.983
Smoking status								
Never smoker					Ref.			
Former smoker					0.87	0.54	1.41	.564
Current smoker					1.10	0.58	2.08	.772
Personal history of cancer (excluding CRC)								
No					Ref.			
Yes					0.90	0.56	1.47	.683
Perception of CRC risk vs average person								
Less likely					Ref.			
About as likely					1.11	0.75	1.63	.603
More likely					1.15	0.49	2.70	.748
Parental history of CRC								
No					Ref.			
Yes					1.59	0.66	3.82	.304

Abbreviation: aOR = adjusted odds ratio; CRC = colorectal cancer; FIT, fecal immunochemical test; FOBT, fecal occult blood test. Significance of bold = *P* < .05.

a Controlled for previous colonoscopy or sigmoidoscopy.

b Sociodemographic factors only, as predictors.

c Sociodemographic factors + CRC risk factors, as predictors.

#### 3.2.2 Model 2 (sociodemographic plus CRC risk factors)

When CRC risk factors were added into the sociodemographic model (Table 2), correlates remained significant (*P* < .05). When comparing in‐office versus home FOBT/FIT, Hispanics were more likely than non‐Hispanics to report in‐office FOBT/FIT (aOR: 2.04, 95% CI: 1.04‐3.99). Respondents 70‐75 years old were less likely than those 50‐59 years old to report in‐office FOBT/FIT (aOR: 0.44, 95% CI: 0.25‐0.79). Additionally, participants residing in the Western US had the lowest odds of using FOBT/FIT in a physician's office (aOR: 0.26, 95% CI: 0.11‐0.58). It is important to note that CRC risk factors such as history of polyps, ulcerative colitis/Crohn disease, alcohol drinking, smoking status, personal history of cancer, and family history of CRC were not significantly associated with CRC screening with FOBT/FIT in the office setting versus at home.

## 4 DISCUSSION

Only 10.4% of age‐eligible adults in our study used FOBT/FIT for CRC screening within the past year. While this represents a slight increase from recent trends in FOBT uptake,^7^ screening using FOBT/FIT in the US remains remarkably low. More than one quarter of respondents reported having been tested in a physician's office in the past year, confirming our main hypothesis. This finding mirrors surveys of physicians that report high rates of in‐office use of FOBT for CRC screening;^18^, ^19^, ^20^, ^31^ however, our study is the first to document in‐office screening prevalence from consumer/patient stand‐point, using population‐based data.

Several factors may account for the dismally low rates of FOBT/FIT uptake amid rising rates of colonoscopy. Among these, physicians' preference for colonoscopy as a screening modality is well documented.^36^ Physicians recommend colonoscopy at a disproportionately higher rate than stool‐based tests, sometimes times without consideration of patient preference.^36^, ^37^ This may drive a higher demand for colonoscopy. Regardless of modality, the ultimate goal—CRC screening—is of essence; however, the availability of multiple effective CRC screening modalities presents a unique opportunity for shared decision making. Training physicians to identify this opportunity and to intentionally propose FOBT/FIT and colonoscopy in equal proportions has been shown to be useful.^38^

Changes to health care policies over the years, regarding preventive services may also have contributed to the widely disparate rates of uptake between colonoscopy and stool‐based tests. With the removal of cost barriers associated with screening, as afforded by the Affordable Care Act, rates of CRC screening, particularly with colonoscopy, increased.^39^ A study comparing CRC screening prevalence before and after the Affordable Care Act among privately and Medicare‐insured adults noted that in some demographic groups, declines in FOBT uptake corresponded with colonoscopy uptake increments, indicative of significant migration from FOBT use to colonoscopy for CRC screening.^39^

Our results also confirmed our hypothesis that sociodemographic factors are correlated with uptake of FOBT/FIT, at home or in‐office. Consistent with previous studies of CRC screening,^24^, ^40^, ^41^, ^42^ our comparison of respondents who have had any FOBT/FIT (regardless of setting) with those who had never carried out these tests found that both sociodemographic factors and CRC risk factors predicted FOBT/FIT uptake (Appendix). However, we showed, for the first time, that situational differences (in‐office vs in‐home) in FOBT/FIT uptake are associated with sociodemographic factors alone and not CRC risk factors. The sociodemographic factors implicated were age, ethnicity, and region. This implies that the relationship between these factors and use of FOBT/FIT holds constant, regardless of setting, even in the presence of co‐existing CRC risk factors and even after controlling for other CRC screening methods (eg colonoscopy and sigmoidoscopy).

A key finding of the current study was a higher odds of CRC screening with in‐office FOBT/FIT among Hispanics. This finding agrees with other studies that have shown Hispanics to have lower odds of screening with FOBT at home^25^, ^28^, ^43^, ^44^, ^45^; therefore, our finding may provide some explanation for the consistently poor rates of home‐based FOBT uptake in this population. Low level of education has previously been identified as a barrier to the use of home‐based FOBT.^45^ Because more than 60% of Hispanics in our study had an education level at or below high school completion, we speculate that low levels of education may have contributed to our finding of an increased likelihood of in‐office as opposed to home‐based FOBT/FIT among Hispanics. This postulation is supported by a study based in an underserved Hispanic population that found higher rates of home‐based FOBT uptake in the intervention arm that had an educational component.^46^ Nevertheless, it is important to note that in‐office screening is mostly physician‐driven; hence it will be useful to learn if certain characteristics among Hispanics predispose them to in‐office screening.

Mirroring previously published work on CRC screening using FOBT,^28^, ^45^ we found that as age increased, so did the likelihood of carrying out FOBT/FIT at home. The referent group in our study (50‐59 years) had a heightened risk of in‐office screening with FOBT/FIT, possibly because it is usually in the 50‐ to 59‐year age bracket that most chronic diseases first manifest and most adults establish regular primary care contact. This contact may include initial counselling about routine screening for CRC. Also, with advancing age and repeated participation, individuals become more accustomed to appropriate screening practices, such as the use of FOBT/FIT at home.

Geographic variations in FOBT/FIT use were also observed. Respondents in the Western region of the country had increased odds of using home‐based FOBT/FIT, with more than 40% of the population who used home tests residing in the West. This guideline‐consistent practice may be attributable to factors ranging from physician recommendations—a previous study showed higher rates of screening compliance in regions where a higher proportion of physician administered screening recommendations^47^—to patient level‐characteristics and public health CRC screening programs. Successful FOBT/FIT‐based, population‐level CRC screening programs have been documented in some states in the West, such as those of the integrated health systems of Kaiser Permanente of Northern California and Group Health of Seattle.^48^ But because the US census regions are geographically and sociodemographically heterogeneous, further studies at smaller geographic units such as state, county, or census tracts would be required to gain further insight into this finding.

Because home‐based FOBT/FIT is recommended by the USPSTF and superior to in‐office testing,^16^ the location of FOBT/FIT uptake has an impact on screening efficacy and guideline compliance. Furthermore, our findings, which identify factors associated with in‐office screening, may be useful in designing targeted interventions, such as those modelled under patient navigation—to improve home‐based uptake of FOBT/FIT. Among other characteristics, patient navigation services are centered on the identification of individual patient‐level barriers to accessing cancer care and prevention services.^49^

Among the adults eligible for CRC screening who had not used FOBT/FIT in the past year, more than 40% had also never had a colonoscopy or sigmoidoscopy. This finding is indicative of marked gaps in the overall uptake of CRC screening. In their longitudinal cohort study, Mandel et al demonstrated the efficacy of FOBT in reducing the incidence of CRC and strongly promoted its use as a population‐level intervention.^15^, ^50^ Therefore, current gaps in the uptake of CRC screening could be addressed by using FOBT/FIT in widespread public health programs. Some population‐based interventions have found FOBT/FIT to be particularly effective in boosting CRC screening uptake when kits are mailed to homes along with instructions and educational packages.^46^, ^48^, ^51^ Similarly, a systematic review revealed that outreach interventions including those utilizing mailed FOBT kits improved CRC screening in Canada, and several European countries.^52^ This initiative should be tested for potential scalability at the national level. Interventions are also needed at the level of care providers, given our finding of continued in‐office screening and other studies pointing to knowledge gaps among health care providers.^20^, ^31^ Hence, educational programs that provide up‐to‐date guideline information and best practices pertinent to CRC screening should be offered to physicians as part of continuing medical education. At the population level, sustained implementation of educational programs may be beneficial, given that previous studies that utilized interventions such as trained community peer educators^46^ and mailed educational kits,^52^ documented improved screening rates.

It is important to acknowledge the limitations of our study. First, the NHIS variable that collects information on stool‐based testing combines FOBT and FIT. While both tests have the same diagnostic outcome and are based on fecal samples, there are important differences between the two tests. FOBT requires multiple stool samples and may require dietary modifications to avoid false‐positive test results. For example FOBT may falsely read positive for hemoglobin from sources other than human blood, for example, red meat. In contrast, FIT has improved sensitivity, as it is designed specifically to detect human hemoglobin arising from the lower gastrointestinal tract.^4^ Nevertheless, current national guidelines promote the use of both tests in the home setting.^4^, ^11^ Second, data collected in NHIS are self‐reported, which are not as reliable as medical/laboratory reports^53^ and prone to recall bias. However, in analyzing only individuals who had FOBT/FIT within the past year, we expect that this recency of screening would enhance recollection of events and thus reduce bias.^54^ Third, physicians play a significant role in in‐office FOBT/FIT use, but data on provider characteristics are unavailable.

Despite these limitations, a key strength of our study is that our sample was restricted to individuals who carried out FOBT/FIT as part of routine care. This ensured we assessed only individuals using FOBT/FIT for screening purposes. In so doing, we addressed a limitation of an earlier study.^7^ Also, the NHIS is a nationally administered survey and provides information that is representative of the US population. Last, the NHIS is administered by trained personnel, and the data are collected in a scientifically rigorous manner.

Amid low overall uptake rates of FOBT/FIT in the US, in‐physician office testing is high, indicative of a missed opportunity for effective CRC screening and poor adherence of physicians to national guidelines. Furthermore, this study describes disparities in the uptake of FOBT/FIT in physicians' office vs at home, highlighting the need to better understand underlying barriers to and promoters of screening in both settings. Our findings point to a number issues that should be further explored. First, further investigations are needed to understand the interactions that occur between the health care provider and patient that ultimately leads to in‐office screening, particularly in minorities such as Hispanics. Also, it is important to evaluate for barriers to home‐based FOBT/FIT in populations with increased rates of in‐office FOBT/FIT. Lastly, since provider adherence to evidence‐based screening practices is crucial for effective CRC prevention and control, interventions aimed at health care providers could potentially bring about declines in in‐office testing rates.

## CONFLICT OF INTEREST

None declared.

## AUTHOR CONTRIBUTIONS

Chido‐Amajuoyi and Shete contributed to study concept and design. Chido‐Amajuoyi and Talluri contributed to data analysis. Chido‐Amajuoyi, Sharma, Tami‐Maury, and Shete contributed to interpretation of the data. Chido‐Amajuoyi and Sharma contributed to initial draft. All authors contributed to critical revision of the manuscript for important intellectual content. Shete obtained funding.

## Data Availability

The data that supports the findings of this study are openly available at https://www.cdc.gov/nchs/nhis/index.htm.
