# Supplementary material for: Physician‐office vs home uptake of colorectal cancer screening using FOBT/FIT among screening‐eligible US adults
Source: Cancer Med. 2019 Oct 21;8(17):7408–18. doi: 10.1002/cam4.2604 (PMC6885889; doi:10.1002/cam4.2604)
Supplement: Supplementary file 1 [file CAM4-8-7408-s001.pdf]

Appendix A. Multivariable logistic regression assessing the association between any use of FOBT/FIT in-office vs. No FOBT/FIT (base outcome) and sociodemographic characteristics and CRC risk factors of US adults (50-75 Years) - National Health Interview Survey, 2015

Predictors	aOR[a,b]	[95% Conf.	Interval]	*P*
**Sex**				
Female	REF			
Male	1.18	0.96	1.46	0.106
**Race**				
White	REF			
Black/African American	1.41	1.05	1.90	**0.021**
American Indian/ Alaska Native	1.67	0.72	3.89	0.232
Asian	1.24	0.81	1.91	0.329
Multiple	0.82	0.33	2.02	0.668
**Hispanic**				
No	REF			
Yes	1.09	0.80	1.48	0.590
**Age, Years**				
50-59	REF			
60-69	1.31	1.04	1.64	**0.019**
70-75	1.74	1.31	2.30	**<0.001**
**Region**				
Northeast	REF			
Midwest	0.73	0.51	1.06	0.102
South	1.08	0.79	1.49	0.615
West	2.30	1.70	3.11	**<0.001**
**Marital Status**				
Not Married	REF			
Married/Living with Partner	1.23	0.99	1.54	0.063
**Highest Education**				
No High School Diploma/ GED Receipient	REF			
High School Graduate	0.99	0.70	1.39	0.944
AA Degree/Some College	0.90	0.66	1.22	0.481
Bachelor's Degree And Higher	1.02	0.75	1.41	0.880
**Health Insurance**				
Not Covered	REF			
Covered	2.27	1.28	4.05	**0.005**
**History of Polyps**				
No	REF			
Yes	1.33	1.05	1.69	**0.018**
**History of Ulcerative Colitis/Crohns Disease**				
No	REF			
Yes	1.25	0.55	2.89	0.592
**Alcoohol Drinking Status**				
Lifetime Abstainer	REF			
Former	1.33	0.96	1.84	0.086
Current (Light-Moderate)	1.14	0.86	1.51	0.349
Current (Heavy)	1.87	1.13	3.10	**0.015**
**Smoking Status**				
Never Smoker	REF			
Former Smoker	1.12	0.89	1.39	0.330
Current Smoker	0.71	0.52	0.97	**0.033**
**Personal History of Cancer (Excluding CRC)**				
No	REF			
Yes	1.40	1.07	1.84	**0.015**
**Perception of CRC Risk Vs. Average Person**				
Less Likely	REF			
About As Likely	0.87	0.71	1.05	0.147
More Likely	0.69	0.46	1.04	0.073
**Parental History of CRC**				
No	REF			
Yes	0.70	0.45	1.11	0.130

[a]controlled for previous colonoscopy or sigmoidoscopy

[b]aOR= adjusted odds ratio

CRC = Colorectal Cancer
